# Integrated Multimodal Critical Care Ultrasound for Mechanism-Based Prediction of Weaning Failure: A Prospective Pilot Study †

**DOI:** 10.3390/jcm15124648

**Published:** 2026-06-15

**Authors:** Şule Asri, Ferhat Soykan, Mustafa Ay, Dilara Tüfek Oztan

**Affiliations:** 1 Intensive Care Unit, Antalya Training and Research Hospital, 07100 Antalya, Türkiye; mustafaayidil@hotmail.com (M.A.); tufekdilara@gmail.com (D.T.O.); 2 Intensive Care Unit, Sincan Training and Research Hospital, 06230 Ankara, Türkiye; ferhatsoykan@gmail.com

**Keywords:** weaning failure, mechanical ventilation, critical care ultrasound, diaphragm ultrasound, VExUS, lung ultrasound, echocardiography, extubation outcome

## Abstract

**Background:** Weaning from mechanical ventilation remains a complex and failure-prone process, with extubation failure rates reaching up to 30%. Conventional indices inadequately capture the multifactorial physiology underlying weaning failure. This study aimed to evaluate whether a multimodal ultrasound approach could improve the identification of mechanisms and prediction of extubation outcomes. **Methods:** In this prospective pilot observational study, adult mechanically ventilated patients with preserved left ventricular ejection fraction (LVEF ≥ 50%) undergoing spontaneous breathing trials (SBT) were included. Multimodal ultrasound assessment—including transthoracic echocardiography (TTE), lung ultrasound (LUS), diaphragmatic ultrasound (DUS), and venous excess ultrasound (VExUS)—was performed at two predefined time points. Conventional respiratory mechanics parameters were recorded concurrently. The primary outcome was a composite of SBT failure (permanent weaning failure) or reintubation within 48 h. **Results:** A total of 27 patients were included, of whom 8 (29.6%) experienced extubation failure (5 permanent SBT failure, 3 post-extubation reintubation). Respiratory system compliance showed consistent associations with extubation failure across both ROC and regression analyses (AUC 0.806, 95% CI 0.611–1.000; cutoff ≤ 45 mL/cmH_2_O; sensitivity 88%; specificity 74%; NPV 93%). Diaphragm excursion was significantly lower in the failure group (*p* = 0.042) and showed useful predictive performance (AUC 0.750, 95% CI 0.565–0.935; cutoff ≤ 24 mm; sensitivity 100%; specificity 58%; NPV 100%). Lung ultrasound, VExUS, and echocardiographic parameters did not demonstrate significant predictive value. Given the limited number of outcome events (*n* = 8) and events-per-variable ratio of 4.0 (EPV = 4.0), all multivariable findings are hypothesis-generating. **Conclusions:** In this prospective pilot study, respiratory system compliance and diaphragm excursion were associated with extubation failure in patients with preserved left ventricular function, while echocardiographic indices, LUS, and VExUS grading did not demonstrate significant predictive value. These hypothesis-generating findings suggest that impaired diaphragmatic function and reduced compliance may be more closely associated with weaning failure than cardiopulmonary congestion parameters. However, given the small sample size, low EPV, and single-centre design, all findings require validation in larger multicentre studies including patients with impaired systolic function.

## 1. Introduction

Liberation from mechanical ventilation represents a critical milestone in the management of critically ill patients yet remains a complex and failure-prone process [1]. Weaning accounts for a substantial proportion of total ventilation time and is associated with increased morbidity and mortality when prolonged [2,3].

Weaning failure is inherently multifactorial, reflecting the interplay between respiratory mechanics, cardiovascular function, diaphragmatic performance, and fluid status [4]. Cardiovascular dysfunction has been traditionally emphasised, with studies suggesting that up to 40% of failed weaning attempts may be related to cardiac causes [5,6,7]. However, conventional bedside indices provide only limited insight into the underlying mechanisms.

Critical care ultrasonography has emerged as a powerful bedside tool for real-time physiological assessment [8]. Individual modalities—including TTE, LUS, and DUS—have demonstrated clinical utility. Lung ultrasound has been shown to correlate with aeration loss and extubation outcomes [9,10], while fluid overload contributes to weaning difficulty, often inadequately detected by conventional methods [11,12]. Diaphragmatic dysfunction is highly prevalent among mechanically ventilated patients and has been associated with weaning outcome [13,14].

More recently, the VExUS grading system has been introduced to assess systemic venous congestion [15,16]. Although diaphragm ultrasound, lung ultrasound, and echocardiography have been individually evaluated in the context of weaning assessment, their systematic integration within a unified, mechanism-based framework remains to be prospectively examined. In particular, VExUS—a structured tool for the bedside assessment of systemic venous congestion—has not previously been incorporated into a multimodal weaning assessment protocol. The present pilot study was therefore undertaken to assess whether the simultaneous application of these four modalities can identify mechanism-specific contributors to weaning failure, and to generate hypotheses for future multicentre studies with adequate statistical power.

The aim of this pilot study was to explore the role of integrated multimodal ultrasound in predicting extubation failure and identifying underlying physiological mechanisms in critically ill patients. The present study extends our preliminary findings reported at the WICC 2023 Congress [17].

## 2. Materials and Methods

### 2.1. Study Design and Setting

This prospective pilot observational study was conducted in the adult ICU of a tertiary-care teaching hospital. The study was conducted in accordance with the Declaration of Helsinki and approved by the Ethics Committee of Antalya Training and Research Hospital (Protocol code: 2022-364, date of approval: 12 January 2023). The study was registered at ClinicalTrials.gov (NCT05763134). Written informed consent was obtained from all patients or their legal representatives prior to enrolment. The study adhered to the STROBE guidelines.

### 2.2. Patient Selection

Consecutive adult patients (≥18 years) receiving invasive MV for ≥48 h and deemed clinically ready for SBT were included. Patients with LVEF < 50% were excluded for two reasons. First, in the context of a pilot study designed primarily to explore the contributions of diaphragmatic function, respiratory mechanics, lung aeration as assessed by ultrasound, and systemic venous congestion as assessed by VExUS grading, the inclusion of patients with systolic dysfunction may have introduced an additional cardiovascular mechanism of weaning failure—namely cardiac-mediated fluid redistribution—potentially obscuring the associations of primary interest.

Second, restricting enrolment to patients with preserved LVEF ensured a more homogeneous study population, which is appropriate in a hypothesis-generating investigation where mechanistic signal detection is prioritised over generalisability. We acknowledge that this selection criterion limits the applicability of the findings to the broader ICU population and that the additive value of echocardiography and VExUS within an unselected multimodal weaning assessment framework remains to be established.

Additional exclusion criteria: neuromuscular disease; high spinal cord injury; significant valvular disease; poor acoustic window; do-not-resuscitate status.

### 2.3. Weaning Protocol and Outcome Definition

All patients underwent a standardised weaning protocol. The SBT consisted of a 30 min trial of pressure support ventilation with positive end-expiratory pressure (PEEP) ≤ 5 cmH_2_O and pressure support ≤10 cmH_2_O. SBT failure was defined by the occurrence of any of the following predefined criteria: SpO_2_ < 90% or PaO_2_/FiO_2_ < 200 mmHg; RR > 35 breaths/min; HR or BP change >20% from baseline; new arrhythmia; accessory muscle use with paradoxical abdominal motion; or altered consciousness. Extubation was performed following successful SBT completion.

The primary outcome was a composite of: permanent SBT failure (requiring continued ventilatory support) or reintubation within 48 h. Although both components reflect an inability to achieve sustained liberation from mechanical ventilation, they represent clinically distinct scenarios: SBT failure occurs prior to extubation and reflects an inability to sustain spontaneous breathing under supported conditions, whereas post-extubation reintubation reflects respiratory decompensation following a successful SBT. The composite endpoint was pre-specified given the small sample size and the overlapping pathophysiological mechanisms underlying both events. Of the 8 patients who experienced the primary outcome, 5 had permanent SBT failure and 3 required reintubation within 48 h. One patient receiving preventive NIV without reintubation was classified as successful extubation.

The rapid shallow breathing index (RSBI), defined as the ratio of respiratory rate to tidal volume (f/VT; breaths/min/L), was measured during the first minute of the SBT. Conventional respiratory mechanics—including static compliance, plateau pressure, and driving pressure—were measured under controlled ventilation immediately prior to SBT initiation.

### 2.4. Ultrasound Protocol

All ultrasound examinations were performed using a CX-50 (Philips Healthcare, Andover, MA, USA) and Vivid-i™ (GE Healthcare, Chicago, IL, USA) by a single operator—the principal investigator, a POCUS-certified intensivist with more than 10 years of dedicated critical care ultrasound experience—thereby ensuring methodological consistency and precluding interobserver variability as a source of measurement error. Examinations were performed at two predefined time points: immediately before SBT initiation (T0) and 30 min into the SBT (T1). All clinical decisions regarding weaning and extubation were made independently of ultrasound findings.

Diaphragm ultrasound was performed with the patient in the semi-recumbent position (head-of-bed elevation 30–45°). The right hemidiaphragm was assessed using a convex or phased-array probe—selected according to the individual patient’s acoustic window—positioned in the subcostal or anterior subcostal view. Diaphragm excursion was measured in M-mode as the maximum cranio-caudal displacement during a complete respiratory cycle. Diaphragm thickening fraction (DTF) was measured using a high-frequency linear probe at the zone of apposition and calculated as the percentage increase in diaphragm thickness from end-expiration to end-inspiration: DTF (%) = [(Tei − Tee)/Tee] × 100, where Tei and Tee represent diaphragm thickness at end-inspiration and end-expiration, respectively. All diaphragm measurements were obtained in triplicate and the mean value was used for analysis.

Lung aeration was assessed using a standardised 12-zone protocol, with each zone scored from 0 (normal aeration) to 3 (consolidation), yielding a global LUS score of 0–36. Echocardiographic assessment included left ventricular ejection fraction, mitral inflow velocities (E/A ratio), and tissue Doppler-derived E/e′ ratio, obtained in accordance with current guideline recommendations. Venous congestion was graded using the VExUS system, incorporating inferior vena cava diameter and Doppler flow patterns of the hepatic, portal, and intrarenal veins [16].

### 2.5. Data Collection

All data were prospectively recorded using a standardised electronic case report form. Variables included baseline characteristics, ventilator settings, SBT parameters, and ultrasound measurements. Complete data were available for all primary ultrasound and respiratory mechanics variables in the 27 included patients, with no missing values requiring imputation.

### 2.6. Statistical Analysis

Continuous variables were assessed for normality using the Shapiro–Wilk test. Normally distributed variables were compared using the independent samples *t*-test; Levene’s test was applied to assess equality of variances, and Welch’s correction was used when variances were unequal. Non-normally distributed variables were compared using the Mann–Whitney U test. Data are presented as mean ± standard deviation or median [interquartile range], as appropriate. Categorical variables are presented as number (percentage) and were compared using Pearson’s Chi-Square test, Fisher’s Exact test, or the Fisher-Freeman-Halton test according to expected cell frequencies.

Receiver operating characteristic (ROC) curves were constructed separately for ultrasound-derived parameters and conventional respiratory parameters. Area under the curve (AUC) confidence intervals were calculated using the DeLong method; sensitivity and specificity confidence intervals were calculated using the Wilson score method. Optimal cutoff values were identified by the Youden index.

Binary logistic regression was performed for all candidate variables. Given the limited number of outcome events (*n* = 8), a parsimonious multivariable model was constructed incorporating the two variables with the strongest a priori physiological rationale—diaphragm excursion and respiratory system compliance—resulting in an events-per-variable ratio (EPV) of 4.0. A second exploratory model was additionally adjusted for age as a potential confounder (EPV = 2.7). As both models fall below the recommended EPV threshold of 10, all multivariable results are considered hypothesis-generating. Analyses were performed using SPSS version 23.0 (IBM Corp., Armonk, NY, USA); a two-tailed *p* < 0.05 was considered statistically significant.

## 3. Results

### 3.1. Study Population and Primary Outcome

A total of 27 mechanically ventilated ICU patients were enrolled, of whom 19 (70.4%) achieved successful extubation and 8 (29.6%) experienced extubation failure. Among the patients in the failure group, 5 experienced permanent SBT failure requiring continued ventilatory support and 3 required reintubation within 48 h of extubation. One patient who received preventive non-invasive ventilation following extubation but did not ultimately require reintubation was classified as having achieved successful extubation. Baseline characteristics and clinical outcomes are presented in Table 1 and Table 2, respectively.

Patients with extubation failure group were significantly older (70.0 ± 18.1 vs. 53.8 ± 15.9 years; *p* = 0.029). APACHE II scores and the duration of mechanical ventilation prior to the SBT did not differ significantly between groups (39.9 ± 4.3 vs. 35.4 ± 11.0, *p* = 0.311; and 5.5 [3.8–9.8] vs. 4.0 [3.0–6.0] days, *p* = 0.319, respectively). Simple weaning was observed exclusively in the successful extubation group (*p* < 0.001). Tracheostomy and ICU mortality were significantly higher in the failure group (50% vs. 5.3%, *p* = 0.017; 62.5% vs. 0%, *p* = 0.001, respectively).

In a pre-specified sensitivity analysis restricted to patients who required post-extubation reintubation (*n* = 3) versus those successfully extubated (*n* = 19), the direction of the associations for both compliance and diaphragm excursion remained consistent with the primary analysis, although statistical significance was not achieved owing to the markedly reduced event number (compliance: 42.0 [41.5–43.5] vs. 52.0 [44.0–62.0] mL/cmH_2_O, *p* = 0.165; diaphragm excursion: 22.9 [20.7–23.4] vs. 24.8 [20.4–29.4] mm, *p* = 0.408).

### 3.2. Conventional Respiratory Parameters

Among conventional respiratory mechanics parameters, compliance was significantly lower in the extubation failure group (39.6 ± 11.0 vs. 53.7 ± 13.8 mL/cmH_2_O; *p* = 0.017), and driving pressure was significantly higher (14.5 ± 3.7 vs. 11.8 ± 1.9 cmH_2_O; *p* = 0.020). Plateau pressure showed a borderline difference that did not reach statistical significance (19.9 ± 4.5 vs. 17.4 ± 2.2 cmH_2_O; *p* = 0.063). RSBI was significantly higher in the failure group (63.8 ± 33.6 vs. 28.9 ± 14.1 breaths/min/L; *p* = 0.022). P0.1, negative inspiratory force, and P/F ratio did not differ significantly between groups (all *p* > 0.05). Detailed parameters are presented in Table 3.

### 3.3. Ultrasound-Derived Parameters

Among ultrasound-derived variables, diaphragm excursion was significantly lower in the extubation failure group (19.8 ± 3.7 vs. 25.1 ± 6.5 mm; *p* = 0.042). Diaphragm thickening fraction showed a consistent trend toward lower values in the failure group that did not reach statistical significance (36.8 ± 21.0 vs. 49.2 ± 17.1%; *p* = 0.118). Lung ultrasound score (20.8 ± 5.4 vs. 18.6 ± 7.0; *p* = 0.443), E/A ratio (1.4 ± 0.7 vs. 1.3 ± 0.6; *p* = 0.700), E/e′ ratio (10.4 [8.4–11.6] vs. 8.8 [6.9–11.5]; *p* = 0.541), and VExUS grade (0.5 [0.0–1.0] vs. 1.0 [0.0–1.5]; *p* = 0.550) did not differ significantly between groups. Fluid balance on the day of extubation showed a borderline trend toward more positive values in the failure group (600 [475–990] vs. 230 [−700–500] mL; *p* = 0.056). All ultrasound-derived parameters are presented in Table 3.

### 3.4. Logistic Regression Analysis

In univariable logistic regression, compliance (OR 0.900, 95% CI 0.814–0.994; *p* = 0.037), driving pressure (OR 1.492, 95% CI 1.001–2.222; *p* = 0.049), and age (OR 1.064, 95% CI 1.001–1.131; *p* = 0.045) were each significantly associated with extubation failure. Diaphragm excursion, despite being significantly lower in the failure group on between-group comparison (*p* = 0.042), did not reach statistical significance in univariable logistic regression (OR 0.836, 95% CI 0.694–1.008; *p* = 0.061), a discrepancy most plausibly attributable to the limited statistical power inherent to a small sample with few outcome events. Lung ultrasound score, VExUS grade, E/A ratio, and E/e′ ratio were not associated with extubation failure in univariable analysis (all *p* > 0.05).

In a parsimonious multivariable model incorporating diaphragm excursion and compliance (EPV = 4.0; Nagelkerke R^2^ = 0.532), compliance retained a statistically significant association with extubation failure (OR 0.886, 95% CI 0.795–0.986; *p* = 0.027), while diaphragm excursion demonstrated a borderline association (OR 0.767, 95% CI 0.583–1.009; *p* = 0.058). In a second exploratory model additionally adjusted for age (EPV = 2.7; Nagelkerke R^2^ = 0.661), compliance remained significant (OR 0.865, 95% CI 0.754–0.993; *p* = 0.039), whereas the associations for diaphragm excursion and age were attenuated and no longer statistically significant (*p* = 0.084 and *p* = 0.087, respectively), consistent with the statistical instability expected under low EPV conditions. As both models fall well below the recommended EPV threshold of 10, all multivariable findings should be regarded as hypothesis-generating. Full regression results are presented in Table 4.

### 3.5. ROC Analysis

**Figure 1:**
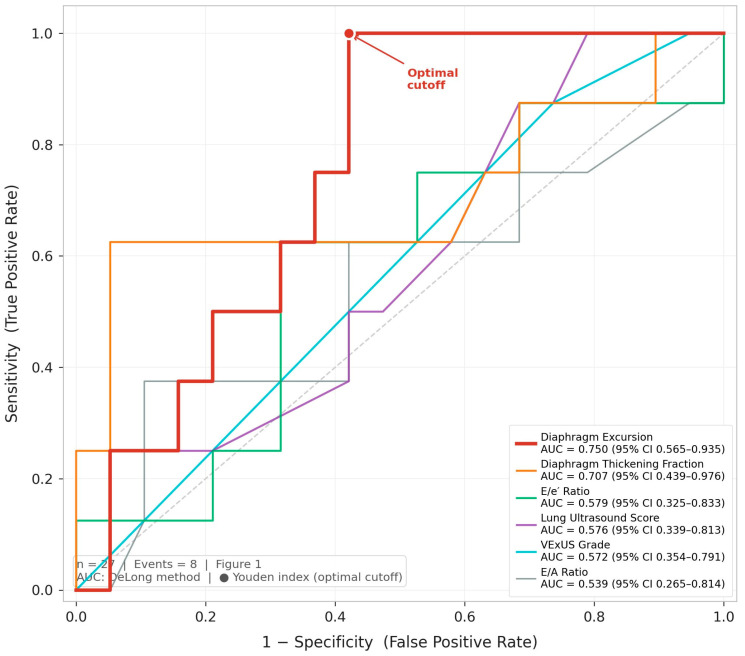
ROC Curves for Ultrasound-Derived Parameters. Diaphragm excursion (AUC 0.750), DTF (0.707), E/e′ (0.579), LUS (0.576), VExUS (0.572), E/A (0.539). Filled circle: Youden optimal cutoff (≤24 mm). Diagonal dashed: AUC = 0.50. AUC CI: DeLong method. *n* = 27; events = 8.

**Figure 2:**
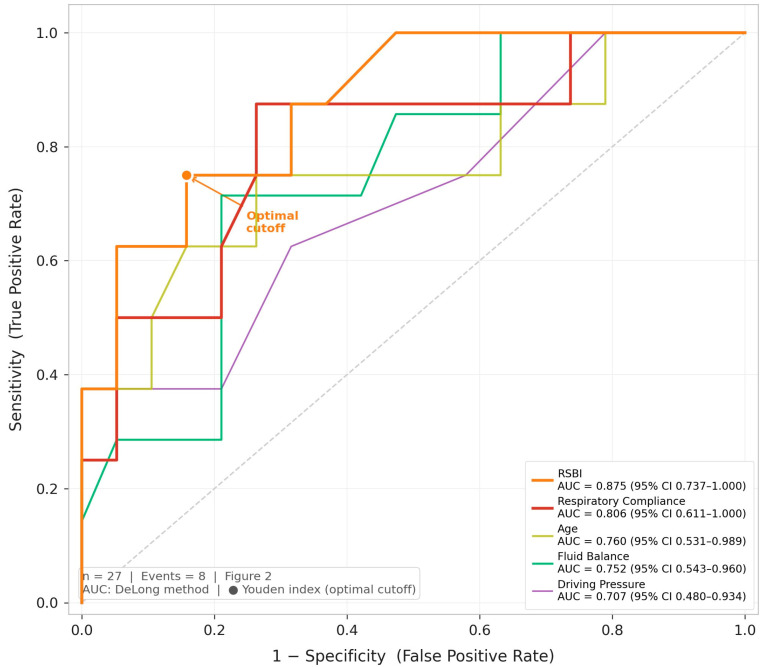
ROC Curves for Conventional Respiratory Parameters. RSBI (AUC 0.875), Compliance (0.806), Age (0.760), Fluid Balance (0.752), Driving Pressure (0.707). Filled circle: Youden optimal cutoff (≤45 mL/cmH_2_O). Diagonal dashed: AUC = 0.50. AUC CI: DeLong method. *n* = 27; events = 8.

Full ROC statistics for ultrasound-derived and conventional parameters are presented in Table 5 and illustrated in Figure 1 and Figure 2, respectively. Among conventional parameters, RSBI demonstrated the highest AUC, while respiratory system compliance showed the most consistent association with extubation failure across ROC and regression analyses (AUC 0.806, 95% CI 0.611–1.000; optimal cutoff ≤ 45 mL/cmH_2_O; sensitivity 88% [95% CI 53–98%]; specificity 74% [95% CI 51–88%]; PPV 58%; NPV 93%). Among ultrasound-derived parameters, diaphragm excursion showed the best predictive performance (AUC 0.750, 95% CI 0.565–0.935; optimal cutoff ≤ 24 mm; sensitivity 100% [95% CI 68–100%]; specificity 58% [95% CI 36–77%]; NPV 100%). Age (AUC 0.760, 95% CI 0.531–0.989) and fluid balance (AUC 0.752, 95% CI 0.543–0.960) demonstrated moderate predictive performance. Diaphragm thickening fraction exhibited high specificity at its optimal cutoff (≤28%: specificity 95% [95% CI 75–99%]; PPV 83%), albeit with limited sensitivity (62% [95% CI 31–86%]). Lung ultrasound score, VExUS grade, E/e′ ratio, and E/A ratio demonstrated limited predictive performance (AUC range 0.539–0.579). Given the small sample size and limited number of events, all confidence intervals are necessarily wide and reported cutoff values should be considered exploratory.

## 4. Discussion

This prospective pilot study evaluated the role of integrated multimodal ultrasound—encompassing diaphragm ultrasound, lung ultrasound, echocardiography, and VExUS grading—in predicting extubation failure among mechanically ventilated patients with preserved left ventricular function. Respiratory system compliance showed consistent associations with extubation failure across analyses (AUC 0.806), while diaphragm excursion was the only ultrasound-derived parameter to show a statistically significant between-group difference (AUC 0.750). Echocardiographic indices, lung ultrasound score, and VExUS grading did not reach statistical significance. These findings are consistent with a physiological framework in which, in the absence of systolic dysfunction, impaired diaphragmatic reserve and increased respiratory load may contribute more substantially to weaning failure than cardiopulmonary congestion in this patient population.

### 4.1. Diaphragm Excursion and Respiratory Mechanics

Diaphragm excursion was significantly lower in the failure group (19.8 ± 3.7 vs. 25.1 ± 6.5 mm; *p* = 0.042). At a cutoff of ≤24 mm, diaphragm excursion demonstrated high sensitivity and NPV (100%). These findings align with the literature showing that diaphragmatic dysfunction has been linked to weaning failure in prior studies [13,14]. It is noteworthy that, despite a significant between-group difference, diaphragm excursion did not reach statistical significance in univariable logistic regression (OR 0.836, 95% CI 0.694–1.008; *p* = 0.061). This discrepancy is most plausibly attributable to the limited statistical power inherent to a small sample with few outcome events, rather than reflecting a true absence of association.

Respiratory system compliance showed the most consistent association with extubation failure across ROC and regression analyses in both univariable (OR 0.900; *p* = 0.037) and multivariable analysis (OR 0.886; *p* = 0.027), with an AUC of 0.806 and a negative predictive value of 93% at an optimal cutoff of ≤45 mL/cmH_2_O. Driving pressure was also significantly associated with failure in univariable analysis (OR 1.492; *p* = 0.049), supporting the association between increased respiratory mechanical load and difficult weaning [18]. Taken together, these findings suggest that successful weaning may depend on the physiological balance between diaphragmatic capacity and respiratory mechanical load.

### 4.2. Interpretation of Negative Findings: LUS, VExUS, and Echocardiography

The absence of significant associations for lung ultrasound score, VExUS grading, and echocardiographic indices warrants careful contextual interpretation and should not be construed as evidence that these modalities lack clinical utility in weaning assessment more broadly. Three interacting study-specific factors most plausibly account for these negative findings.

First, the SBT was conducted using pressure support ventilation with PEEP ≤ 5 cmH_2_O and pressure support ≤10 cmH_2_O—a relatively supported modality compared with T-piece or minimal-assistance trials. PSV-based SBTs are known to attenuate the haemodynamic challenge of the weaning attempt by limiting the reduction in intrathoracic pressure, the rise in venous return, and the increase in left ventricular afterload [5]. Under these conditions, echocardiographic parameters reflecting diastolic filling pressures and VExUS congestion indices are unlikely to change substantially, even in patients who might ultimately fail a more demanding unassisted trial. This interpretation is supported by evidence from Moschietto et al. and de Almeida et al. demonstrating that echocardiographic diastolic indices can identify cardiovascular-mediated weaning failure [19,20], and by Cabello et al. showing that T-piece trials impose substantially greater haemodynamic stress than PSV-based SBTs, rendering cardiac dysfunction signals more detectable [5].

Second, the LVEF ≥ 50% inclusion criterion by design excluded patients in whom cardiac dysfunction is a major contributor to weaning failure. In a cohort without systolic impairment, echocardiographic and VExUS parameters are expected to demonstrate lower predictive performance, as the pathophysiological substrate they are designed to detect is largely absent. This selection effect is a fundamental contextual consideration when interpreting the negative echocardiographic and VExUS findings [6,7], and it substantially limits the generalisability of these results to the broader ICU population.

Third, limited statistical power cannot be excluded as a contributing factor. The consistently low AUC values for echocardiographic and LUS parameters (range 0.539–0.579) suggest that these modalities did not demonstrate clear prognostic signal within this specific cohort, a finding that most likely reflects the interplay of the above-mentioned design constraints rather than an inherent inability to predict weaning outcome. This interpretation is consistent with evidence suggesting that echocardiographic and congestion-related parameters may provide greater predictive utility in populations with underlying cardiac dysfunction [6,7] and that diaphragmatic indices show directionally consistent associations with difficult weaning in more demanding protocols [18]. Notably, diaphragm thickening fraction showed a directionally consistent trend with these prior reports (36.8 ± 21.0 vs. 49.2 ± 17.1%; *p* = 0.118; AUC 0.707), and statistical significance may have been achievable in a larger, less selected cohort. Indeed, a post hoc power calculation indicates that approximately 45–60 patients (20–25 outcome events) would be required to achieve 80% power to detect the observed magnitude of DTF difference, underscoring the need for adequately powered future studies.

### 4.3. Age as a Potential Confounder

Patients in the extubation failure group were significantly older than those who were successfully extubated (70.0 ± 18.1 vs. 53.8 ± 15.9 years; *p* = 0.029), and age itself demonstrated moderate predictive performance (AUC 0.760). Advanced age may serve as a surrogate for overall physiological reserve, encompassing reduced respiratory muscle mass, diminished neural drive, impaired mucociliary clearance, and greater comorbidity burden—all of which may contribute to weaning failure independently of diaphragmatic function and respiratory system compliance [1,13]. While the limited event number precluded definitive adjustment, the consistency of the compliance association across both the unadjusted and age-adjusted multivariable models provides partial reassurance that the observed association is not entirely attributable to age-related confounding.

### 4.4. Limitations

This study has several important limitations that must be considered when interpreting its findings. First, the small sample size (*n* = 27, 8 events) substantially restricts the reliability of multivariable logistic regression, with an EPV of 4.0 in the primary model—well below the commonly recommended threshold of 10; all multivariable findings should accordingly be regarded as hypothesis-generating [21]; a post hoc power calculation further indicates that approximately 45–60 patients (20–25 outcome events) would be required to achieve 80% power to detect the observed magnitude of DTF difference. Second, the PSV-based SBT modality likely attenuated the haemodynamic challenge of the weaning attempt and may have reduced the detectability of cardiac-mediated weaning failure signals; future studies should incorporate T-piece or minimal-support trials to more fully characterise the cardiovascular dimension of weaning physiology [5]. Third, the LVEF ≥ 50% inclusion criterion limits the generalisability of the findings to the broader ICU population and may have contributed to the negative echocardiographic and VExUS findings, as cardiovascular dysfunction is more commonly implicated in weaning failure among patients with impaired systolic function [6,7]. Accordingly, the additive value of these modalities within an unselected multimodal weaning assessment framework remains to be established [22]. Fourth, the significant age difference between groups represents a potential confounder that could not be fully accounted for given the limited event number. Finally, the single-centre design and the restriction to patients with preserved systolic function limit the external validity and generalisability of the present findings; confirmation in larger, multicentre cohorts encompassing the full spectrum of left ventricular function is warranted.

## 5. Conclusions

In this prospective pilot study of mechanically ventilated patients with preserved left ventricular function, diaphragm excursion and respiratory system compliance were associated with extubation failure, while echocardiographic indices, lung ultrasound score, and VExUS grading did not demonstrate significant predictive value. These findings suggest that, in patients without systolic dysfunction, impaired diaphragmatic function and reduced respiratory system compliance may contribute more substantially to weaning failure than cardiopulmonary congestion parameters in this patient population. However, given the small sample size, low events-per-variable ratio, and single-centre design, all findings should be regarded as hypothesis-generating. The role of integrated multimodal ultrasound—particularly the additive value of VExUS—warrants evaluation in larger, adequately powered multicentre studies including patients with reduced ejection fraction.

## Figures and Tables

**Table 1:** Baseline characteristics.

Variable	Overall (*n* = 27)	Successful (*n* = 19)	Failure (*n* = 8)	*p*-Value
**Demographics**				
**Age, years**	**58.6 ± 17.9**	**53.8 ± 15.9**	**70.0 ± 18.1**	**0.029**
Male sex, *n* (%)	11 (40.7%)	7 (36.8%)	4 (50.0%)	0.675
Medical admission, *n* (%)	19 (70.4%)	15 (78.9%)	4 (50.0%)	0.183
**Illness Severity**				
APACHE II score	36.6 ± 9.8	35.4 ± 11.0	39.9 ± 4.3	0.311
GCS at ICU admission	13.0 [8.0–15.0]	14.0 [7.5–15.0]	12.0 [9.8–15.0]	0.978
MV duration prior to SBT, days	4.0 [3.0–6.0]	4.0 [3.0–6.0]	5.5 [3.8–9.8]	0.319
P/F ratio at T0, mmHg	326.8 ± 94.3	331.2 ± 101.0	316.5 ± 81.5	0.720
**Comorbidities**				
COPD, *n* (%)	5 (18.5%)	5 (26.3%)	0 (0.0%)	0.280
Diabetes mellitus, *n* (%)	13 (48.1%)	9 (47.4%)	4 (50.0%)	0.999
Chronic renal failure, *n* (%)	8 (29.6%)	5 (26.3%)	3 (37.5%)	0.658
Active neoplasm, *n* (%)	7 (25.9%)	5 (26.3%)	2 (25.0%)	0.999
Haematological malignancy, *n* (%)	5 (18.5%)	3 (15.8%)	2 (25.0%)	0.616
Immunosuppression, *n* (%)	7 (25.9%)	5 (26.3%)	2 (25.0%)	0.999
Chronic liver failure, *n* (%)	3 (11.1%)	3 (15.8%)	0 (0.0%)	0.532
Atrial fibrillation, *n* (%)	1 (3.7%)	0 (0.0%)	1 (12.5%)	0.296
**Fluid Status and Haemodynamics at T0**				
Fluid balance on extubation day, mL	410.0 [−578–660]	230.0 [−700–500]	600.0 [475–990]	0.056

Data are presented as mean ± standard deviation for normally distributed continuous variables, and as median [interquartile range] for non-normally distributed variables; categorical variables are presented as number (percentage). Normality was assessed using the Shapiro–Wilk test. Continuous variables were compared using the independent samples *t*-test, with Welch’s correction applied when Levene’s test indicated unequal variances, or the Mann–Whitney U test for non-normally distributed variables. Categorical variables were compared using Fisher’s exact test or the Fisher–Freeman–Halton test. Statistically significant *p*-values (*p* < 0.05) are shown in bold. Abbreviations: APACHE: Acute Physiology and Chronic Health Evaluation; GCS: Glasgow Coma Scale; MV: mechanical ventilation; P/F: PaO_2_/FiO_2_ ratio; COPD: chronic obstructive pulmonary disease; T0: immediately before spontaneous breathing trial initiation; SBT: spontaneous breathing trial.

**Table 2:** Clinical outcomes.

Variable	Overall (*n* = 27)	Successful (*n* = 19)	Failure (*n* = 8)	*p*-Value
**Weaning Category**				
**Simple weaning, *n* (%)**	**16 (59.3%)**	**16 (84.2%)**	**0 (0.0%)**	**<0.001**
Difficult weaning, *n* (%)	2 (7.4%)	2 (10.5%)	0 (0.0%)	
Permanent SBT failure, *n* (%)	5 (18.5%)	0 (0.0%)	5 (62.5%)	
Post-extubation reintubation, *n* (%)	3 (11.1%)	0 (0.0%)	3 (37.5%)	
Preventive NIV ^†^, *n* (%)	1 (3.7%)	1 (5.3%)	0 (0.0%)	
**ICU Outcomes**				
**Tracheostomy, *n* (%)**	**5 (18.5%)**	**1 (5.3%)**	**4 (50.0%)**	**0.017**
**ICU mortality, *n* (%)**	**5 (18.5%)**	**0 (0.0%)**	**5 (62.5%)**	**0.001**
**ICU survival, *n* (%)**	**22 (81.5%)**	**19 (100.0%)**	**3 (37.5%)**	**0.001**
**Respiratory Status at ICU Discharge ^‡^**				
**No oxygen therapy, *n* (%)**	**10 (45.5%)**	**10 (52.6%)**	**0 (0.0%)**	**0.014**
Oxygen therapy, *n* (%)	10 (45.5%)	9 (47.4%)	1 (33.3%)	
Tracheostomy/invasive MV, *n* (%)	2 (9.1%)	0 (0.0%)	2 (66.7%)	

Data are presented as number (percentage). Categorical variables were compared using Fisher’s exact test or the Fisher–Freeman–Halton test. Statistically significant *p*-values (*p* < 0.05) are shown in bold. ^†^ This patient received preventive non-invasive ventilation following extubation but did not require reintubation and was therefore classified as having achieved successful extubation per the primary outcome definition. ^‡^ Respiratory status at ICU discharge is reported only for patients surviving to ICU discharge: all 19 patients in the successful extubation group survived; 3 of 8 patients in the extubation failure group survived to ICU discharge (5 died in the ICU). Percentages for discharge status are calculated among survivors in each group. Abbreviations: SBT: spontaneous breathing trial; NIV: non-invasive ventilation; MV: mechanical ventilation; ICU: intensive care unit.

**Table 3:** Conventional respiratory mechanics and ultrasound-derived parameters.

Variable	Overall (*n* = 27)	Successful (*n* = 19)	Failure (*n* = 8)	*p*-Value
**Conventional Respiratory Mechanics (T0, controlled ventilation)**				
**Static compliance, mL/cmH_2_O**	**49.6 ± 14.4**	**53.7 ± 13.8**	**39.6 ± 11.0**	**0.017**
Plateau pressure, cmH_2_O	18.1 ± 3.2	17.4 ± 2.2	19.9 ± 4.5	0.063
**Driving pressure, cmH_2_O**	**12.6 ± 2.8**	**11.8 ± 1.9**	**14.5 ± 3.7**	**0.020**
**RSBI ^†^, breaths/min/L**	**39.2 ± 26.6**	**28.9 ± 14.1**	**63.8 ± 33.6**	**0.022**
P0.1, cmH_2_O	2.0 [1.0–2.8]	2.0 [1.0–3.0]	1.0 [1.0–2.1]	0.271
Negative inspiratory force, cmH_2_O	−14.6 ± 7.2	−15.5 ± 7.3	−12.4 ± 7.0	0.320
P/F ratio during SBT	326.8 ± 94.3	331.2 ± 101.0	316.5 ± 81.5	0.720
**Diaphragmatic Ultrasound**				
**Diaphragm excursion at SBT, mm**	**23.5 ± 6.3**	**25.1 ± 6.5**	**19.8 ± 3.7**	**0.042**
Diaphragm thickening fraction (T0), %	45.5 ± 18.8	49.2 ± 17.1	36.8 ± 21.0	0.118
**Lung Ultrasound**				
LUS score (0–36, T0)	19.2 ± 6.6	18.6 ± 7.0	20.8 ± 5.4	0.443
**Echocardiography**				
LVEF, %	55.6 ± 5.4	56.1 ± 5.9	54.4 ± 4.2	0.474
E/A ratio (T0)	1.4 ± 0.6	1.3 ± 0.6	1.4 ± 0.7	0.700
E/e′ ratio (T0)	9.4 [7.3–11.5]	8.8 [6.9–11.5]	10.4 [8.4–11.6]	0.541
**VExUS Grading**				
VExUS grade (0–3, T0)	1.0 [0.0–1.0]	1.0 [0.0–1.5]	0.5 [0.0–1.0]	0.550
VExUS 0, *n* (%)	12 (44.4%)	8 (42.1%)	4 (50.0%)	0.999
VExUS 1, *n* (%)	9 (33.3%)	6 (31.6%)	3 (37.5%)	
VExUS 2, *n* (%)	5 (18.5%)	4 (21.1%)	1 (12.5%)	
VExUS 3, *n* (%)	1 (3.7%)	1 (5.3%)	0 (0.0%)	
**Fluid Status**				
Fluid balance, mL	410 [−578–660]	230 [−700–500]	600 [475–990]	0.056

Data are presented as mean ± standard deviation for normally distributed continuous variables, and as median [interquartile range] for non-normally distributed variables; categorical variables are presented as number (percentage). Normality was assessed using the Shapiro–Wilk test. Continuous variables were compared using the independent samples *t*-test, with Welch’s correction applied when Levene’s test indicated unequal variances, or the Mann–Whitney U test for non-normally distributed variables. Categorical variables were compared using Fisher’s exact test or the Fisher–Freeman–Halton test. Statistically significant *p*-values (*p* < 0.05) are shown in bold. ^†^ RSBI (rapid shallow breathing index) was measured during the first minute of the SBT and is defined as the ratio of respiratory rate to tidal volume (f/VT; breaths/min/L). Abbreviations: RSBI: rapid shallow breathing index; P/F: PaO_2_/FiO_2_ ratio; LUS: lung ultrasound score; LVEF: left ventricular ejection fraction; E/A: mitral early-to-atrial peak velocity ratio; E/e′: ratio of mitral early peak velocity to tissue Doppler early diastolic annular velocity; VExUS: venous excess ultrasound; T0: immediately before SBT initiation; SBT: spontaneous breathing trial.

**Table 4:** Logistic regression analysis.

Variable	B	SE	Wald	OR (95% CI)	*p*-Value
**UNIVARIABLE LOGISTIC REGRESSION**					
**Compliance (mL/cmH_2_O)**	**−0.105**	**0.051**	**4.27**	**0.900 (0.814–0.994)**	0.037 *
**Driving pressure (cmH_2_O)**	**0.400**	**0.203**	**3.87**	**1.492 (1.001–2.222)**	0.049 *
**Age (years)**	**0.062**	**0.031**	**4.01**	**1.064 (1.001–1.131)**	0.045 *
Diaphragm excursion (mm)	−0.179	0.095	3.52	0.836 (0.694–1.008)	0.061 ^†^
Plateau pressure (cmH_2_O)	0.267	0.163	2.69	1.307 (0.949–1.799)	0.101
DTF (%)	−0.042	0.027	2.35	0.959 (0.909–1.012)	0.126
E/e′ ratio	0.068	0.073	0.87	1.071 (0.927–1.236)	0.352
LUS score	0.055	0.070	0.63	1.057 (0.921–1.212)	0.429
VExUS grade	−0.389	0.529	0.54	0.678 (0.240–1.912)	0.463
E/A ratio	0.272	0.678	0.16	1.313 (0.348–4.957)	0.688
**MULTIVARIABLE MODEL 1—DE + Compliance (EPV = 4.0; Nagelkerke R^2^ = 0.532)**					
**Compliance (mL/cmH_2_O)**	**−0.122**	**0.055**	**4.88**	**0.886 (0.795–0.986)**	0.027 *
Diaphragm excursion (mm)	−0.265	0.140	3.58	0.767 (0.583–1.009)	0.058 ^†^
**MULTIVARIABLE MODEL 2—Age-adjusted (EPV = 2.7; Nagelkerke R^2^ = 0.661)**					
**Compliance (mL/cmH_2_O)**	**−0.145**	**0.070**	**4.27**	**0.865 (0.754–0.993)**	0.039 *
Diaphragm excursion (mm)	−0.266	0.154	2.99	0.766 (0.567–1.036)	0.084 ^†^
Age (years)	0.081	0.047	2.94	1.084 (0.988–1.190)	0.087 ^†^

Univariable logistic regression was performed for all candidate variables. Given the limited number of outcome events (*n* = 8), a parsimonious multivariable Model 1 was constructed incorporating the two variables with the strongest a priori physiological rationale—diaphragm excursion and respiratory system compliance—yielding an events-per-variable ratio (EPV) of 4.0. Model 2 additionally adjusted for age as a potential confounder (EPV = 2.7). As both models fall below the commonly recommended EPV threshold of 10, all multivariable findings are considered hypothesis-generating and should be interpreted with caution. The term “independent predictor” is deliberately avoided. Analyses were performed using SPSS version 23.0 (IBM Corp., Armonk, NY, USA). Abbreviations: B: regression coefficient; SE: standard error; Wald: Wald chi-square statistic; OR: odds ratio; CI: confidence interval; EPV: events-per-variable ratio; DTF: diaphragm thickening fraction; LUS: lung ultrasound score; VExUS: venous excess ultrasound; E/A: mitral early-to-atrial peak velocity ratio; E/e′: ratio of mitral early peak velocity to tissue Doppler early diastolic annular velocity. * *p* < 0.05; ^†^ *p* < 0.10.

**Table 5:** Receiver Operating Characteristic (ROC) analysis.

Variable	Cutoff	AUC (95% CI)	Sensitivity (95% CI)	Specificity (95% CI)	PPV	NPV
**Ultrasound-Derived Parameters (Figure 1)**						
Diaphragm excursion (mm)	≤24	0.750 (0.565–0.935)	100% (68–100%)	58% (36–77%)	50%	100%
DTF (%)	≤28	0.707 (0.439–0.976)	62% (31–86%)	95% (75–99%)	83%	86%
E/e′ ratio	≥9.9	0.579 (0.325–0.833)	62% (31–86%)	68% (46–85%)	45%	81%
LUS score	≥12	0.576 (0.339–0.813)	100% (68–100%)	21% (9–43%)	35%	100%
VExUS grade	—	0.572 (0.354–0.791)	—	—	—	—
E/A ratio	—	0.539 (0.265–0.814)	—	—	—	—
**Conventional Parameters (Figure 2)**						
RSBI (breaths/min/L)	≥41	0.875 (0.737–1.000)	75% (41–93%)	84% (62–94%)	67%	89%
Compliance (mL/cmH_2_O)	≤45	0.806 (0.611–1.000)	88% (53–98%)	74% (51–88%)	58%	93%
Age (years)	≥63	0.760 (0.531–0.989)	75% (41–93%)	74% (51–88%)	55%	88%
Fluid balance (mL)	≥550	0.752 (0.543–0.960)	71% (36–92%)	79% (57–91%)	56%	88%
Driving pressure (cmH_2_O)	≥17	0.707 (0.480–0.934)	38% (14–69%)	100% (83–100%)	100%	79%

AUC confidence intervals calculated using the DeLong method. Sensitivity and specificity confidence intervals calculated using the Wilson score method. Optimal cutoff values determined by the Youden index. For variables with AUC < 0.60, no clinically meaningful cutoff is reported. PPV: positive predictive value; NPV: negative predictive value. All estimates are exploratory given the small sample size (*n* = 27, 8 events); reported cutoff values should not be applied clinically without validation in larger cohorts. Green shading: AUC ≥ 0.75. Abbreviations: AUC: area under the curve; CI: confidence interval; PPV: positive predictive value; NPV: negative predictive value; RSBI: rapid shallow breathing index (f/VT; breaths/min/L); DTF: diaphragm thickening fraction; LUS: lung ultrasound score; VExUS: venous excess ultrasound; E/A: mitral early-to-atrial peak velocity ratio; E/e′: ratio of mitral early peak velocity to tissue Doppler early diastolic annular velocity.

## Data Availability

Available upon reasonable request from the corresponding author.
